# A Comparative Study on Alvogyl and a Mixture of Black Seed Oil and Powder for Alveolar Osteitis: A Randomized Double-Blind Controlled Clinical Trial

**DOI:** 10.1155/2022/7756226

**Published:** 2022-02-28

**Authors:** Zafar Ali Khan, Namdeo Prabhu, Naseer Ahmed, Abhishek Lal, Rakhi Issrani, Afsheen Maqsood, Fahim Vohra, Mohammad Khursheed Alam

**Affiliations:** ^1^Department of Oral and Maxillofacial Surgery and Diagnostic Sciences, College of Dentistry, Jouf University, Sakaka, Saudi Arabia; ^2^Department of Prosthodontics, Altamash Institute of Dental Medicine, Karachi 75500, Pakistan; ^3^Prosthodontics Unit, School of Dental Sciences, Health Campus, Universiti Sains Malaysia, 16150 Kubang Kerian, Kota Bharu, Kelantan, Malaysia; ^4^Department of Preventive Dentistry, College of Dentistry, Jouf University, Sakaka, Al Jouf 72345, Saudi Arabia; ^5^Department of Oral Pathology, Bahria University Dental College, Karachi 75530, Pakistan; ^6^Department of Prosthetic Dental Science, College of Dentistry, King Saud University, Riyadh, Saudi Arabia; ^7^Center for Transdisciplinary Research (CFTR), Saveetha Dental College, Saveetha Institute of Medical and Technical Sciences, Saveetha University, Chennai 600077, India; ^8^Department of Public Health, Faculty of Allied Health Sciences, Daffodil International University, Dhaka 1207, Bangladesh

## Abstract

**Introduction:**

Alveolar osteitis (AO) is the most common complication faced by exodontia patients and is usually seen 24–74 hours after tooth extraction, heralded by severe throbbing pain. *Nigella sativa* is commonly known as black seed known to have anti-inflammatory and antibacterial properties along with other reparative properties that enhance bone formation. This study aimed to evaluate and compare the effects of Alvogyl and a mixture of *Nigella sativa* powder and oil in the treatment of dry sockets.

**Materials and Methods:**

Sixty patients above the age of 18 and below 70 years, from both genders, who underwent extraction of teeth and were clinically diagnosed with a dry socket at the clinic of the College of Dentistry, Jouf University, Saudi Arabia, were included in this study. Pain scores were assessed after placement of the dressing at the following intervals: 5 minutes, 30 minutes, 60 minutes, 2^nd^ day, 4^th^ day, and 7^th^ day. Patients were randomly allocated to three groups, namely, Group 1 (Alvogyl), Group 2 (mixture of *Nigella sativa*'s powder and oil), and Group 3 (control). Pain relief and healing of the socket were compared between the three groups. The collected data were subjected to statistical analysis through Spearman's correlation test, independent *t*-test, ANOVA, and post hoc test.

**Results:**

A mixture of *Nigella sativa* powder and oil showed a statistically significant difference in relieving pain compared to the Alvogyl group. A mixture of *Nigella sativa*'s powder and oil required fewer dressings when compared to the Alvogyl group.

**Conclusion:**

A mixture of *Nigella sativa* powder and oil is the more efficacious dressing material for the management of dry sockets compared to Alvogyl. It provides immediate and complete pain relief and fewer numbers of repeated visits.

## 1. Introduction

Alveolar osteitis or “dry socket” is the most common complication faced by patients following tooth extraction, usually seen after 24–74 hours [1, 2]. The entity was labeled as ‘dry socket' or ‘alveolar osteitis' in 1896 by Crawford and is defined as an acute inflammation of the alveolar bone observed after two to four days around the site of the extracted tooth socket heralded by severe throbbing pain and the disintegration of the blood clot formed inside the extraction socket that is often filled with food debris [3–5].

Several previous studies reported diverse incidences of dry socket ranging from 5 to 30% in surgical removal of impacted mandibular third molars and between 1 and 4% in simple extraction cases of permanent teeth [6, 7]. The exact cause of dry socket is not precisely understood. Nevertheless, numerous denominators have been implicated, including age, gender, oral contraceptive use, tobacco cigarette smoking, tooth location, bone and soft tissue injury from problematic extractions, poor oral hygiene, and volume of vasoconstriction administered along with local anesthetics used, operative and postoperative management, and operator skill [2].

The local fibrinolytic activity is implicated as the main reason leading to dry socket [6]. The surge in fibrinolytic activity after extraction may subsequently lead to premature disintegration of the intraalveolar clot [2]. The fibrinolysis is the consequence of plasminogen pathway activation, initiated via direct physiologic activators after alveolar osteoid cells or secondary activator substances secreted by bacteria [8].

The exact pathogenesis of dry socket in association with tobacco cigarette smoking is to date unidentified. The largely accepted and dominant hypothesis suggests that the sucking motion associated with cigarette smoking causes mechanical clot dislodgement [2, 3].

The treatment of this painful condition is wide-ranging including washing and flooding the empty tooth socket with a copious amount of normal saline and or placement of intrasocket medication, namely, Alvogyl, zinc oxide eugenol, chlorhexidine, metronidazole, olive oil-black seed paste, and honey [9, 10].

Despite the availability of various medications for the treatment of dry-socket pain, not a single treatment option provides immediate and complete relief from symptoms. Subsequently, the tooth socket heals secondarily in ten to fourteen days with variable severity of pain continued throughout this healing period [10, 11]. To enhance the efficacy of healthcare, the World Health Organization (WHO) has encouraged developing countries to integrate the use of therapeutic plants since more than two-thirds of their population depends on the use of natural remedies and traditional herbs for disease treatment [12, 13].

Complementary and alternative medicine (CAM) is the term for medical products and practices that are not part of standard medical care. There is an increasing interest towards the use of CAM in the field of medicine [14, 15]. Patients with a variety of chronic conditions are using CAM to help cope with their disease [16–18]. The CAM products (*Ferula* assa-foetida oleo-gum resin) have been actively used in the field of dentistry to treat and prevent oral diseases related to oral mucosa and dental caries [19, 20]. One such plant is *Nigella sativa* commonly known as black seed, black cumin, black caraway, kalojeera, kalonji, or kalonji [21]. Its chemical composition contains volatile and nonvolatile oils in addition to many other active ingredients including proteins, alkaloids, coumarines, saponins, minerals, carbohydrates, phenolic compounds, and steroidal compounds. Many studies have been conducted on the effects of *Nigella sativa* seed extracts on various body systems in vitro or in vivo. The pharmacological investigation of the seed extracts reveals a broad spectrum of activities including antidiabetic, anti-inflammatory, and osteoreparative properties that enhance bone formation [22].

One of the treatment options that is commonly used to treat patients suffering from the dry socket is Alvogyl. Alvogyl consists of active ingredients such as iodoform (antibacterial) and butamben (anesthetic) [23]. Alvogyl has been successfully studied in patients as it is known to provide relief from pain and discomfort in patients suffering from dry sockets [24]. Furthermore, Alvogyl has been compared with neocone and zinc oxide eugenol, with Alvogyl being superior to both of the agents [25].

Considering the abovementioned there is a need to investigate the efficacy of *Nagilla Sativa* on the postextraction complications of dry socket. To the best of our knowledge, this is the first comparative study of olive oil-black seed oil for the treatment of dry socket at the College of Dentistry, University of Jouf, Kingdom of Saudi Arabia, and is aimed to find out the best and most effective medication for the treatment of dry socket at this institution.

## 2. Materials and Methods

### 2.1. Study Setting, Sample Size, and Subjects Inclusion Criteria

The OpenEpi software was used to calculate the sample size. Considering the mean value of 2.567 ± 2.678 [23] for dry socket pain severity, the power of the test was 80%. A confidence interval (CI) of 95%, and a margin of error of 5%. The estimated sample size for each of the three groups was 20 participants. Therefore, a total of 60 patients were included in the study. In this comparative study, patients were selected from the dental clinics of the College of Dentistry, Jouf University, Sakaka, based on the following predetermined inclusion/exclusion criteria.

#### 2.1.1. Inclusion Criteria

1. Patients over the age of 18, from both genders, who had their teeth extracted and were clinically diagnosed with dry socket

#### 2.1.2. Exclusion Criteria

1. Patients under the age of 18 years and over the age of 70 years
2. Patient with various bone diseases including osteoporosis
3. Patients who had a history of taking oral or intravenous bisphosphonates
4. Patients with a history of radiotherapy to the head and neck and jawbones

### 2.2. Ethical Considerations

Written informed consent was obtained from all patients. The demographic information such as age, gender, and address was documented in a performa. The type of treatment and medication provided was beneficial and nonharmful to the patients, according to medical ethics. The study was conducted according to the guidelines of the Declaration of Helsinki and approved by the Institutional Review Board of Jouf University (ethical review number: 11-04-42). This trial has been registered under the Saudi Clinical Trial Registry (SCTR) with application no. 21021702. The confounding variables such as age, gender, and history of pain were controlled by matching.

### 2.3. Study Design

Patients clinically diagnosed with alveolar osteitis were assigned numbers from first to the sixtieth patient and randomly allocated via lottery to three groups named as follows: (1) Alvogyl (Septodont, Saint-Maur-des-Fosses, France) (2) mixture of *Nigella sativa*'s powder and oil (Kalonji Oil, *Nigella sativa*, Organic Pure Oil, Jeddah, Saudi Arabia), and (3) normal saline rinse as a control. Both the patient and the investigator assessing the treatment outcome were blinded from identifying the medicament dressing used for the group. The medication used was placed in unmarked identical bottles and saline in a covered syringe without the original label on them and marked as 1, 2, and 3.

### 2.4. Outcomes

Patients were allowed to continue their oral analgesic medication, namely, ibuprofen 200 mg, 400 mg, or 600 mg twice daily, depending upon the severity of initial pain upon diagnosis. Pain relief was recorded and compared between the groups on a visual analog pain scale at every appointment. The intraalveolar medication was repeated until the postoperative pain symptoms subsided. Patients were reviewed at 5 minutes, 30 minutes, and 60 minutes after dressing. The patient was requested to note daily pain records on a (0–10) visual analog scale, with zero representing no pain and ten representing the worst pain. The patients were requested to note daily pain records on a (0–10) visual analog scale for 7 days.

They also recorded any harmful effects of medication and advised to immediately call the investigator about any problem in the due course. If no side effects were noted, the treatment option was repeated a maximum of four times over two weeks to assess the complete effect of the medication in case complete relief was not achieved the first time. The total time required for complete healing and the number of repeated sessions for each medication till complete relief from all symptoms were recorded.

### 2.5. Data Analysis

The data was analyzed in SPSS-25 (Statistical Package for Social Sciences, version 25, IBM, Chicago, Illinois). A descriptive analysis was carried out to calculate the mean and standard deviation, percentage, and frequency of quantitative and qualitative variables such as age, gender, scores of the VAS scale, values for different study groups assigned, and the severity of pain categories.

The independent *t*-test was used to check for gender disparities. ANOVA and the post hoc test were used to compare the outcomes of different groups assigned. Spearman's correlation test was used to evaluate the association of age and gender with the mean VAS scores of patients in each group. The *p*value of ≤0.05 was considered statistically significant.

## 3. Results

**Figure 1:**
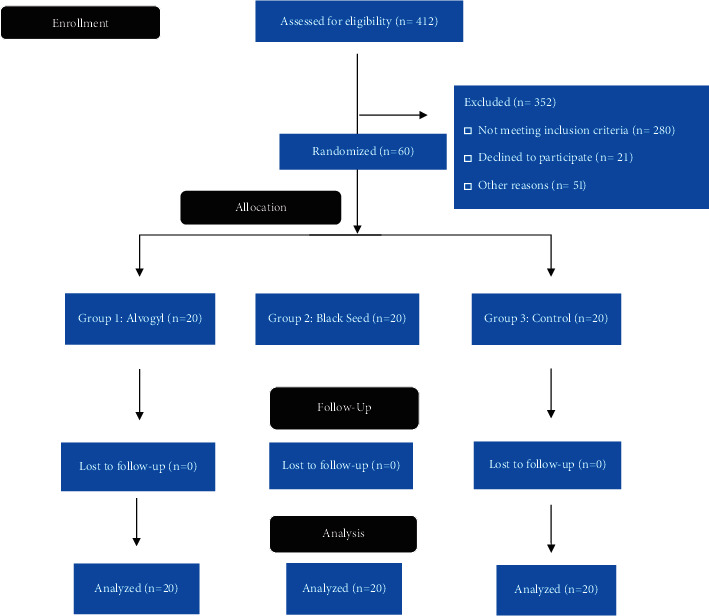
CONSORT flow diagram of the study.

This randomized controlled trial consisted of sixty patients that were randomized into 3 groups as follows: Group 1, Alvogyl; Group 2, black seed; and Group 3, control, as presented in Figure 1.

The mean age of the patients in each group was as follows: Group 1, 41.70; Group 2, 53.15; and Group 3, 49.30. About the gender, the distribution of the males and females in each group was as follows: Group 1, 11 and 9; Group 2, 10 and 10; and Group 3, 8 and 12. As for the comparison of age and gender with VAS pain scores amongst the groups, age and Group 1 patient VAS scores had a significant relationship (*p*value = 0.047), and gender and Group 2 patients also had a significant relationship (*p*value = 0.037), as presented in Table 1.

The mean pain scores of the patients before the placement of the intrasocket in all of the three groups are presented in Table 2. In this study, the incidence of dry socket was found in 16 (26.6%) patients after anterior teeth extractions and 44 (73.3%) patients after posterior teeth extractions in both upper and lower arches. Moreover, 26 (43.3%) of the dry socket cases were found in the maxilla and 44 (56.6%) in the mandible.

**Figure 2:**
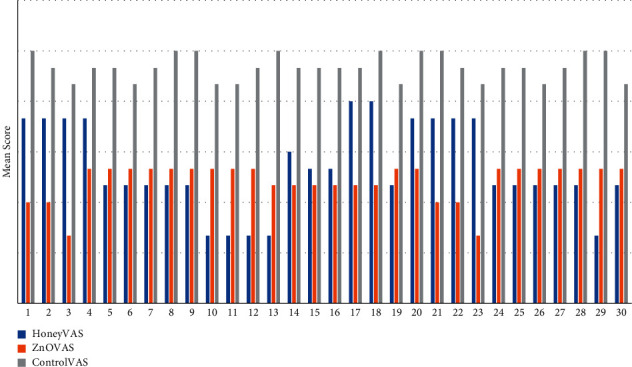
Distribution of mean VAS scores of patients.

The mean postmedication VAS pain score of patients in all the three groups was noted at the following time intervals: 5 minutes postmedication, 30 minutes postmedication, 60 minutes postmedication, 2^nd^ day postmedication, 4^th^ day postmedication, and 7th day postmedication. The majority of patients reported a decrease in pain scores in both groups 1 and 2, respectively, over time, as presented in Figure 2.

The patients of Group 3 did not report a significant decrease in pain at 5 minutes postmedication. At 30 minutes and 60 minutes postmedication, a decrease in pain score was seen in patients belonging to groups 1 and 2, but the decrease was more significant in Group 2. However, patients in Group 3 did not experience a significant decrease in pain at the same interval. From the 2^nd^ day postmedication, all of the patients belonging to Group 2 were pain-free as compared to Group 1 where patients were still experiencing pain. On the 4th day postmedication, patients belonging to Group 1 still suffered minimal pain which disappeared completely in all of the patients. Patients belonging to Group 3 reported minimal to no pain on the 7^th^ day postmedication, as presented in Table 2.

The intergroup comparison through the application of the ANOVA test showed a significant difference between the mean scores of the three groups (A and B, *p*=0.031), (A and C, *p*=0.011), and (B and C, *p*=0.001), respectively, as presented in Table 3. Furthermore, the post hoc test indicated that patients treated with normal saline alone showed a high intensity of dry socket pain (*p*=0.001) as compared to the Alvogyl and *Nigella sativa* powder and oil groups. Additionally, the least VAS scores were found in the Nigella Sativa powder and oil group compared to Alvogyl.

Regarding the gender of the patients, an independent *t*-test was used to evaluate the difference between the VAS scores of males and females. No significant difference was found between the genders of all the groups (*p*value = 0.525), as presented in Table 4.

## 4. Discussion

Alveolar osteitis is one of the most common complications that arise as a postoperative complication associated frequently with the extraction of mandibular third molars. Since the pain of dry socket as experienced by the patients is severe and excruciating, this mandates timely management to relieve the discomfort and pain of the patients. Different risk factors are associated with a greater tendency to develop dry sockets such as smoking, use of oral contraceptives, female gender, suppression of the immune system, and traumatic extractions.

In our study, most of the patients suffered from severe pain using a visual analog scale when they were diagnosed to suffer from dry socket. Such findings have previously been reported in a study by Hawker et al., where patients who were suffering from dry socket reported severe dental pain. Since the bone around the socket is exposed to the oral environment with supplemental action of the bacteria, these factors then lead to the severe pain of the dry socket [26].

Firstly, in our study, patients who were assigned to Alvogyl dressing treatment in the socket reported a mild decrease in pain scores that were still significant, causing patients' pain and discomfort. Such findings have also been reported in a study by Faizel et al., who concluded that patients assigned with Alvogyl dressing experienced a decrease in the level of pain [25]. One of the ingredients of Alvogyl is eugenol which has sedative, antibacterial, and anodyne effects. Moreover, Alvogyl also contains butamben which is an anesthetic substance along with iodoform which is antibacterial [18]. These properties possessed by Alvogyl make it suitable for patients suffering from dry sockets. However, one study by Abdull Gaffar et al. reported that the use of Alvogyl in the sockets of patients resulted in delayed healing and inflammation of the socket [27].

Secondly, in our study, we applied black seed liquid with powder in the sockets of patients suffering from dry sockets. In our study, we found that the pain relief of the patients in this group was significantly less as compared to Alvogyl immediately after its application. Patients belonging to black seed dressing groups were pain-free on the 2nd postmedication day. Dalimunte et al. found that the application of black seed in the sockets of the patients resulted in a significantly higher period of wound healing with 12% black cumin to have the most optimum effect on the healing of the socket [28]. One of the components of black seed is thymoquinone. One study by Syafriadi et al. reports that the use of thymoquinone postextraction sockets of diabetic induced rats resulted in an improvement of reepithelization at the margin of the sockets [29]. Hence, further studies are required to evaluate the use of thymoquinone as a preventive measure in high-risk patients that might develop dry sockets such as diabetic patients and smokers. Furthermore, it has been found that the use of powder of black seed in postextraction cases of patients resulted in more radiopacity with complete disappearance of the lamina dura in a time duration of 6 weeks along with complete epithelization [30].

In our study, the severity of pain between males and females was equal with no significant relationship between them. These results contrast with studies in the literature that report higher severity of pain in females as compared to males [31]. Moreover, the female gender has been known to be a risk factor for the potential development of dry socket [32].

In the literature, different treatment options are available for the treatment of dry socket. Platelet-rich fibrin (PRF) has been used to treat dry sockets with a study reporting an early reduction in pain levels experienced by patients with minimal need of analgesic intake [33]. Moreover, honey has also been used to treat patients with dry sockets due to a significant reduction in inflammation, discomfort, pain, and hyperemia [34, 35]. Other therapies can have a significant influence on the oral environment. The use of ozone and laser photodynamic therapies can modify clinical and microbiological parameters in periodontal patients, and they could also have an effect in the response to the technique described in the present report [36, 37]. As in our study, it was found that a mixture of black seeds oil and powder can be effectively used as an alternative to Alvogyl in patients suffering from dry socket. All these different methods of treatment should be considered in future clinical trials to further evaluate the effectiveness of each of them in treating alveolar osteitis.

Dry socket is a condition that has a wide choice of treatment options such as Alvogyl, honey, zinc oxide eugenol, and black seed. Despite the strengths of this study such as regular follow-up of the patients and reporting of the pain scores, this study was met with some limitations. Firstly, the sample size of the patients in each group was small, and lastly, patients might not have followed the temporary cessation of habits during the healing period of sockets such as smoking.

## 5. Conclusion

The use of Alvogyl resulted in a gradual decrease in pain scores; however, black seed powder with oil resulted in immediate pain relief for the patients, being more effective than Alvogyl and the use of normal saline irrigation alone. Hence, a black seed oil mixture can be used effectively in patients suffering from alveolar osteitis.

## Figures and Tables

**Table 1:** Correlation of age and gender with mean VAS pain scores amongst the study groups.

Variables		Mean VAS pain score
Alvogyl		
Age	Correlation coefficient	−0.450^*∗*^
	*p*value	0.047
Gender	Correlation coefficient	−0.401
	*p*value	0.080

A mixture of black seed oil and powder		
Age	Correlation coefficient	0.183
	*p*value	0.440
Gender	Correlation coefficient	0.468^*∗*^
	*p*value	0.037

Control		
Age	Correlation coefficient	0.343
	*p*value	0.139
Gender	Correlation coefficient	0.417
	*p*value	0.067

**Table 2:** Distribution mean VAS scores of patients pre and postmedication.

Groups		Preoperative VAS pain score	5 mins postmedication VAS pain score	30 minutes postmedication VAS pain score	60 minutes post medication VAS pain score	Second day postmedication VAS pain score	4^th^ day postmedication VAS pain score	7th day post medication VAS pain score
Group A (Alvogyl)	Mean	8.85	5.60	4.55	3.55	2.55	0.85	0.01
	Std. deviation	0.75	0.50	0.51	0.51	0.51	0.74	0.01
Group B (mixture of *Nigella sativa*'s powder and oil)	Mean	8.55	2.40	1.20	0.05	0.01	0.02	0.01
	Std. deviation	1.05	0.59	0.41	0.22	0.02	0.03	0.01
Group C (normal saline rinse as a control)	Mean	7.80	6.70	6.35	6.05	4.40	1.60	0.60
	Std. deviation	1.01	0.57	0.48	0.22	0.50	0.75	0.50

VAS: visual analog scale.

**Table 3:** Comparison of VAS score of patients among the three groups using the ANOVA test.

Groups	*N*	Mean and SD	Group comparison *F*	*p*value
Alvogyl (Group A)	20	3.70 ± 0.50	A and B	0.031
A mixture of *Nigella sativa*'s powder and oil (Group B)	20	1.74 ± 0.33	A and C	0.011
Control (Group C)	20	4.78 ± 0.57	B and C	0.001

**Table 4:** Gender disparities among the patients of all groups, independent *t*-test.

Gender	*N*	Mean	Standard deviation	Standard error mean	Mean difference	*t*-value	df	*p*value
Male	29	2.44	1.51	0.28	−0.25	−0.64	58	0.525
Female	31	2.70	1.58	0.28				

## Data Availability

The raw data used to support the findings of this study are included within the article.

## References

[1] Mohammed H. A. Y. (2011). Dry socket: frequency, clinical picture, and risk factors in a Palestinian dental teaching center. *The Open Dentistry Journal*.

[2] Taberner-Vallverdu M., Nazir M., Sanchez-Garces M., Gay-Escoda C. (2015). Efficacy of different methods used for dry socket management: a systematic review. *Medicina Oral, Patología Oral y Cirugía Bucal*.

[3] Parthasarathi K., Smith A., Chandu A. (2011). Factors affecting incidence of dry socket: a prospective community-based study. *Journal of Oral and Maxillofacial Surgery*.

[4] Cardoso C. L., Rodrigues M. T. V., Júnior O. F., Garlet G. P., de Carvalho P. S. P. (2010). Clinical concepts of dry socket. *Journal of Oral and Maxillofacial Surgery*.

[5] Preetha S. (2014). An overview of dry socket and its management. *IOSR Journal of Dental and Medical Science*.

[6] Blum I. R. (2002). Contemporary views on dry socket (alveolar osteitis): a clinical appraisal of standardization, aetiopathogenesis and management: a critical review. *International Journal of Oral and Maxillofacial Surgery*.

[7] Noroozi A.-R., Philbert R. F. (2009). Modern concepts in understanding and management of the “dry socket” syndrome: comprehensive review of the literature. *Oral Surgery, Oral Medicine, Oral Pathology, Oral Radiology & Endodontics*.

[8] Kolokythas A., Olech E., Miloro M. (2010). Alveolar osteitis: a comprehensive review of concepts and controversies. *International Journal of Dentistry*.

[9] Mamoun J. (2018). Dry socket etiology, diagnosis, and clinical treatment techniques. *Journal of the Korean Association of Oral and Maxillofacial Surgeons*.

[10] Veale B. (2015). Alveolar osteitis: a critical review of the aetiology and management. *Oral Surgery*.

[11] Bloomer C. R. (2000). Alveolar osteitis prevention by immediate placement of medicated packing. *Oral Surgery, Oral Medicine, Oral Pathology, Oral Radiology & Endodontics*.

[12] World Health Organization (2013). *WHO Traditional Medicine Strategy: 2014-2023*.

[13] AlAttas S. A., Zahran F. h. M., Turkistany S. A. (2016). Nigella sativa and its active constituent thymoquinone in oral health. *Saudi Medical Journal*.

[14] Krami R., Hashempur M. H., Tavakoli A. (2017). Effects of Fumaria parviflora L on uremic pruritus in hemodialysis patients: a randomized, double-blind, placebo-controlled trial. *Jundishapur Journal of Natural Pharmaceutical Products*.

[15] Razmgah G. R. G., Hosseini S. M. R., Hajimehdipoor H. (2021). The effect of an aloe-based polyherbal formulation in adults with functional constipation: a randomized, double-blind, placebo-controlled, six-months clinical follow-up trial. *Traditional and Integrative Medicine*.

[16] Trejo A. C., Castañeda I. D., Rodríguez A. C. (2021). Hydrogen peroxide as an adjuvant therapy for COVID-19: a case series of patients and caregivers in the Mexico city metropolitan area. *Evidence-based Complementary and Alternative Medicine*.

[17] Nayebi N., Esteghamati A, Meysamie A (2019). The effects of a Melissa officinalis L. based product on metabolic parameters in patients with type 2 diabetes mellitus: a randomized double-blinded controlled clinical trial. *Journal of Complementary & Integrative Medicine*.

[18] Xu X., Zhang M, Wu X, Zheng C, Huang G (2022). The effect of electroacupuncture treatment with different intensities for functional diarrhea: a randomized controlled trial. *Evidence-based Complementary and Alternative Medicine*.

[19] Seyed Hashemi M., Hashempur M. H., Lotfi M. H. (2019). The efficacy of asafoetida (Ferula assa-foetida oleo-gum resin) versus chlorhexidine gluconate mouthwash on dental plaque and gingivitis: a randomized double-blind controlled trial. *European Journal of Integrative Medicine*.

[20] Liu Y., Meng Y., Wu M., Zhang Q. (2021). A two-year longitudinal study of the effectiveness of the CRT® bacteria test in evaluating caries risk in three-year-old children. *Evidence-based Complementary and Alternative Medicine*.

[21] Mekhemar M., Hassan Y., Dörfer C. (2020). Nigella sativa and thymoquinone: a natural blessing for periodontal therapy. *Antioxidants*.

[22] Soni N., Singh V., Mohammad S. (2016). Effects of honey in the management of alveolar osteitis: a study. *National Journal of Maxillofacial Surgery*.

[23] Kalsi H. K., Major R., Jawad H. (2020). Alvogyl or alveogyl?. *British Dental Journal*.

[24] Supe N., Choudhary S., Yamyar S., Patil K., Choudhary A., Kadam V. (2018). Efficacy of alvogyl (Combination of Iodoform + Butylparaminobenzoate) and zinc oxide eugenol for dry socket. *Annals of Maxillofacial Surgery*.

[25] Faizel S., Thomas S., Yuvaraj V., Prabhu S., Tripathi G. (2015). Comparision between neocone, alvogyl and zinc oxide eugenol packing for the treatment of dry socket: a double blind randomised Control trial. *Journal of Maxillofacial and Oral Surgery*.

[26] Hawker G. A., Mian S., Kendzerska T., French M. (2011). Measures of adult pain: visual analog scale for pain (VAS pain), numeric rating scale for pain (NRS pain), McGill pain questionnaire (MPQ), short-form McGill pain questionnaire (SF-MPQ), chronic pain grade scale (CPGS), short form-36 bodily pain scale (SF. *Arthritis Care & Research*.

[27] AbdullGaffar B. (2016). Alvogyl dental dressing: a potential cause of complicated postextraction non-healing sockets: a clinicopathologic study of 7 cases. *International Journal of Dentistry and Oral Health*.

[28] Dalimunte R. S., Hanafiah O. A., Rusdy H. (2020). The effect of black cumin (nigella sativa sp.) gel extract in wound healing process post tooth extraction. *Journal of Biomimetics, Biomaterials and Biomedical Engineering*.

[29] Syafriadi M., Yusuf P. R., Pratama S. M., Ummah D. Z., Amalia K. (2018). The correlation of consuming thymoquinone extract of nigella sativa to tooth socket re-epithelization on diabetic-induced rats. *Journal of Dentomaxillofacial Science*.

[30] Al-Hijazi A. (2013). Evaluation of the effect of nigella sativa oil and powder on socket healing process. *Journal of Natural Sciences Research*.

[31] Paul S., Choudhury R., Kumari N. (2019). Is treatment with platelet-rich fibrin better than zinc oxide eugenol in cases of established dry socket for controlling pain, reducing inflammation, and improving wound healing?. *Journal of the Korean Association of Oral and Maxillofacial Surgeons*.

[32] Bartley E. J., Fillingim R. B. (2013). Sex differences in pain: a brief review of clinical and experimental findings. *British Journal of Anaesthesia*.

[33] Chakravarthi S. (2017). Platelet rich fibrin in the management of established dry socket. *Journal of the Korean Association of Oral and Maxillofacial Surgeons*.

[34] Rakhshan V. (2015). Common risk factors for postoperative pain following the extraction of wisdom teeth. *Journal of the Korean Association of Oral and Maxillofacial Surgeons*.

[35] Ansari A., Joshi S., Garad A., Mhatre B., Bagade S., Jain R. (2019). A study to evaluate the efficacy of honey in the management of dry socket. *Contemporary Clinical Dentistry*.

[36] Butera A., Gallo S., Pascadopoli M., Luraghi G., Scribante A. (2021). Ozonized water administration in peri-implant mucositis sites: a randomized clinical trial. *Applied Sciences*.

[37] Koochaki M., Hendi A., Ghasemi M., Seyedjafari E., Hamidain M., Chiniforush N. (2021). Comparative evaluation of the effects of antimicrobial photodynamic therapy with an LED and a laser on the proliferation of human gingival fibroblasts on the root surface: an in vitro study. *Journal of Lasers in Medical Sciences*.

